# Plant Sterol Ester of *α*-Linolenic Acid Attenuates Nonalcoholic Fatty Liver Disease by Rescuing the Adaption to Endoplasmic Reticulum Stress and Enhancing Mitochondrial Biogenesis

**DOI:** 10.1155/2019/8294141

**Published:** 2019-12-12

**Authors:** Hao Han, Yan Guo, Xiaoyu Li, Dongxing Shi, Tingli Xue, Linqi Wang, Yanyan Li, Mingming Zheng

**Affiliations:** ^1^Department of Nutrition and Food Hygiene, School of Public Health, Shanxi Medical University, No. 56, Xinjian South Road, Taiyuan, Shanxi 030001, China; ^2^Shenzhen Center for Chronic Disease Control, 2021 Buxin Road, Shenzhen 518020, China; ^3^Oil Crops Research Institute, Chinese Academy of Agricultural Sciences, Hubei Key Laboratory of Lipid Chemistry and Nutrition, Oil Crops and Lipids Process Technology National & Local Joint Engineering Laboratory, Key Laboratory of Oilseeds Processing, Ministry of Agriculture, Wuhan 430062, China

## Abstract

Nonalcoholic fatty liver disease (NAFLD) is becoming more common in the world and is presenting a great challenge concerning prevention and treatment. Plant sterol ester of *α*-linolenic acid (PS-ALA) has a potential benefit to NAFLD. To examine the effect of PS-ALA on NAFLD, C57BL/6J mice were given a control diet, high fat and high cholesterol diet (HFD), and HFD plus 2% PS, 1.3% ALA, or 3.3% PS-ALA for 16 weeks. Our results showed that PS-ALA treatment suppressed hepatic steatosis, ameliorated lipid disorder, attenuated inflammatory response, and inhibited oxidative stress. In the molecular level, PS-ALA downregulated high transcriptional and translational levels of endoplasmic reticulum (ER) stress markers (*Grp78* and *Chop*) leading to decreased protein expression of transcription factor and key enzymes involved in de novo lipogenesis (*Srebp-1c* and *Fas*) and cholesterol synthesis (*Srebp-2* and *Hmgcr*). In parallel, PS-ALA blocked *Nlrp3* activation and reduced release of IL-1*β* and IL-18 via inhibiting ER stress-induced sensitization of unfolded protein response sensors (*Ire1α* and *Xbp1s*). Finally, PS-ALA improved HFD-induced mitochondrial damage and fatty acid accumulation as exhibited by higher protein and mRNA expression of key genes administering mitochondrial biogenesis (*Pgc-1α*, *Nrf1*, and *Tfam*) and fatty acid *β*-oxidation (*Pparα* and *Cpt1a*). In conclusion, our study originally demonstrated that PS-ALA rescued ER stress, enhanced mitochondrial biogenesis, and thus ameliorated NAFLD.

## 1. Introduction

Currently, nonalcoholic fatty liver disease (NAFLD) is becoming the most prevalent chronic liver disease around the world and the main cause of hepatocellular carcinoma (HCC), liver transplantation, and liver-related mortality [1]. According to recent data, NAFLD is affecting 25% of adult people worldwide [2] and 85-98% of obese patients [3]. Although the evidence raises a concern about the impact of NAFLD in the world, the basic mechanism that initiates NAFLD remains elusive [4]. Recently, evidence revealed that endoplasmic reticulum (ER) stress played a crucial role in the development of NAFLD [5]. ER is the main cell organ taking charge of protein folding, lipid biogenesis, and calcium homeostasis. Stressors that impair the folding capacity of the ER may lead to immature defective protein overflows and trigger ER stress [6]. Although ER stress may active a cascade of compensatory responses, called unfolded protein response (UPR), helping restore ER homeostasis and cell survival, persistent ER stress is known to enforce a detrimental pathological outcome, involving ectopic fat deposition, inflammation, oxidative stress, apoptosis, and dysregulated autophagy [7]. All of the processes mentioned above are capable of provoking the development of NAFLD [8].

Moreover, recent data in the literature showed that NAFLD was caused by ER stress-mediated mitochondrial dysfunction [9]. ER stress activates ER oxidoreductin 1 (ERO1) and leads to reactive oxygen species (ROS) overproduction. Then, ROS activates ER protein inositol-1,4,5-trisphosphate receptors and inactivates sarcoplasmic reticulum Ca^2+^-ATPase, which resulted in the raised level of cytosolic Ca^2+^, increased mitochondrial uptake of Ca^2+^, and ultimately mitochondrial dysfunction [10]. Being the primary site for the *β*-oxidation of fatty acid (FA), mitochondria play a central role in FA degradation. Impaired mitochondrial FA *β*-oxidation may result in fat accumulation in the liver and implicate in the pathogenesis of NAFLD [11].

It has been reported that intracellular accumulation of saturated fatty acid (SFA) and cholesterol, seen in obesity or atherosclerosis, resulted in ER stress [12]. Thus, modulation of lipid metabolism through dietary intervention may be capable of ameliorating SFA or cholesterol overload-induced ER stress.

Plant sterol (PS) is a specific phytochemical widely existing in cereal, legumes, vegetables, and fruits. Because of structural similarity to cholesterol, PS has a good effect on lowering cholesterol concentrations by competing with cholesterol for intestinal absorption [13]. Daily intake of 2 g PS is in the recommendations of the national cholesterol education program (NCEP) in decreasing total cholesterol (TC) and low-density lipoprotein cholesterol (LDL-C) level [14]. However, plant sterol was shown to have a limited effect on lowering circulating triglyceride (TG) levels [15]. It has been well established that *α*-linolenic acid (ALA), a plant-based n-3 polyunsaturated fatty acid (PUFA), has a potent effect in lowering TG, increasing FA catabolism, and inhibiting inflammation [16, 17]. Accordingly, plant sterol has been recently esterified by ALA to obtain a final product, plant sterol ester of *α*-linolenic acid (PS-ALA), which would simultaneously reduce TC as well as TG and thus may improve ER homeostasis and protects against NAFLD.

However, the effect of PS-ALA on NAFLD and the exact molecular mechanism are still poorly understood. Therefore, the relationship between PS-ALA intake and chronic high fat and high cholesterol diet- (HFD-) induced NAFLD in mice was explored in this work. Furthermore, the potential molecular mechanism underlying the action of PS-ALA was deeply investigated mainly focusing on ER homeostasis and mitochondrial biogenesis. Understanding the mechanisms by which PS-ALA improves NAFLD may provide additional details on the potential impact of PS-ALA on metabolic function and NAFLD management.

## 2. Materials and Methods

### 2.1. Materials

*α*-Linolenic acid (*α*-linolenic acid 80.67%) was purchased from Henan Linuo Biochemistry Co., Ltd. (Anyang, Henan, China). Plant sterol (*β*-sitosterol 85.04%, campesterol 7.83%, brassicasterol 1.15%, and stigmasterol 1.02%) was purchased from Xian Bluesky Biological Engineering Co., Ltd. (Xian, Shanxi, China). The plant sterol ester of *α*-linolenic acid was synthesized by the Oil Crops Research Institute, Chinese Academy of Agricultural Sciences (Wuhan, China) [18].

### 2.2. Animals and Treatments

50 male C57BL/6J mice were purchased from Beijing Vital River Laboratory Animal Technology Co., Ltd. (Beijing, China) and transferred to the laboratory animal facilities. The experimental protocol was approved by the Research Ethics Committee of Shanxi Medical University, China. After acclimatization for one week, the animals were randomly and equally allocated to five experimental groups. These were the control group (control), high fat and high cholesterol diet (HFD) group, plant sterol (PS) group, *α*-linolenic acid (ALA) group, and plant sterol ester of *α*-linolenic acid (PS-ALA) group. The control group was given a normal diet with 10 kcal% fat. The HFD group was given a high fat and high cholesterol diet with 45 kcal% fat, 20 kcal% protein, 35 kcal% carbohydrate, and 2% cholesterol (*w*/*w*). The PS-ALA group was given the same HFD containing 3.3% PS-ALA. The PS group and the ALA group were given the same HFD containing 2% PS and 1.3% ALA, respectively (equivalent dose of ALA and PS to PS-ALA). The composition of the experimental diets of each group was shown in Table 1.

After 16 weeks, all the mice were fasted for 12 h and then sacrificed. Liver tissue and blood samples were collected for further analysis.

### 2.3. Histology Examination

Paraffin-embedded fixed liver tissue was cut in 5 *μ*m and stained with hematoxylin and eosin (H/E) for histopathological examination. Fresh-frozen liver cross-sections (5 *μ*m) were incubated with Oil Red O solution for lipid staining. The images were captured with an optical microscope (CKX53, Olympus) and analyzed using Image-Pro Plus (IPP) software.

### 2.4. Biochemical Assays

Serum lipid profiles (TC, TG, LDL-C, and HDL-C) and liver lipid content (TC, TG) were examined by enzymatic colorimetric assays using commercially available detection kits (Biosino Biotechnology Co., Ltd., Beijing, China). The level of aspartate aminotransferase (AST) and alanine aminotransferase (ALT) was measured by a Mindray BS-200 automatic biochemistry analyzer (Shenzhen, China) with matching kits.

### 2.5. Enzyme-Linked Immunosorbent Assay (ELISA)

Serum interleukin-1*β* (IL-1*β*) and interleukin-18 (IL-18) were assayed by ELISA under the guidance of the instruction of the commercially available detection kit (R&D Systems, Minneapolis, MN, USA).

### 2.6. ROS Detection

Frozen liver cross-sections (5 *μ*m) were incubated with dihydroethidium (5 mol/L) for 15 min. Then, the fluorescence level was visualized and captured with a fluorescence microscope and quantified using IPP.

### 2.7. Gene Expression Profiling

Total RNA of liver samples was extracted using the TRIzol reagent (Invitrogen, USA) and reverse transcribed to cDNA according to the manufacturer's instructions (TaKaRa Biotechnology Co., Ltd., Dalian, China). The mRNA expression in liver tissue was quantified using the SYBR Green detection system in 7900HT instrument (Applied Biosystems, Forster, CA, USA). The primer sequences are listed in Table 2.

### 2.8. Western Blotting Analysis

Total protein of liver samples was extracted, and the protein concentrations were measured according to the instruction of a BCA Protein Assay Kit (Applygen, Beijing, China). Equal amounts of protein were heated at 98°C with sodium dodecyl sulfate loading buffer and separated by sodium dodecyl sulfate-polyacrylamide gel electrophoresis (SDS-PAGE). The proteins were transferred to the polyvinylidene fluoride (PVDF) membrane and blocked with 5% skimmed milk for 2 h. After incubating with specific primary antibodies overnight at 4°C, the blots were incubated with the horseradish peroxidase- (HRP-) conjugated species-specific second antibodies. Then, the immunoreactive bands were detected by the ECL Detection System (Syngene, Cambridge, UK). Quantitative analysis of the density of the bands was performed with ImageJ software.

### 2.9. Statistical Analysis

The data were analyzed using one-way (ANOVA) with SPSS 20.0 software package and shown as the mean ± standard error of mean (SEM). *P* < 0.05 was considered a statistically significant difference between groups.

## 3. Results

### 3.1. PS-ALA Decreased Liver Wet Weight and Body Weight of Mice

As shown in Table 3, at the end of the experiment, the mice given HFD had greater liver wet weight and body weight than normal mice (*P* < 0.05). PS-ALA treatment significantly decreased the liver wet weight and body weight of HFD-fed mice (*P* < 0.05). However, there was no difference in food intake between each group (*P* > 0.05).

### 3.2. PS-ALA Attenuated Lipid Accumulation in the Liver

**Figure 1:**
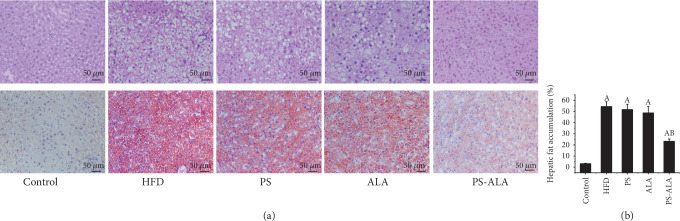
PS-ALA ameliorates hepatic steatosis in mice. (a) H/E and (b) Oil Red O staining of lipid droplets in the livers of mice in each group (magnification ×200). (c) Quantitative analysis of hepatic fat accumulation. Data represents as means ± standard error of the mean and is normalized to % of field area (*n* = 6). ^a^*P* < 0.05 versus the control group; ^b^*P* < 0.05 versus the HFD group.

Histological evaluation of H/E and Oil Red O staining revealed that HFD led to a large quantity of fat accumulation. PS-ALA treatment markedly improved fat degeneration (Figures 1(a) and 1(b)). Quantitative analysis of the extent of adipose infiltration demonstrated a significantly smaller size in PS-ALA-treated animals than that in HFD-treated mice (*P* < 0.05) (Figure 1(c)).

### 3.3. PS-ALA Suppressed HFD-Induced ER Stress

**Figure 2:**
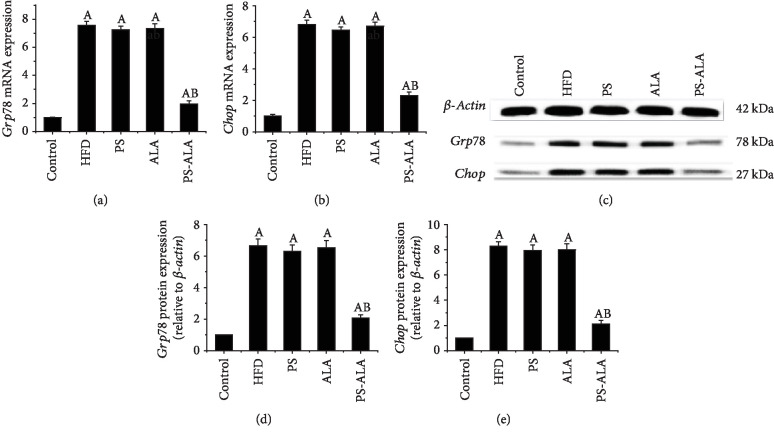
PS-ALA alters protein expression of gene involved in ER stress. Effect of PS-ALA on mRNA expression of (a) *Grp78* and (b) *Chop* was assayed with quantitative real-time RT-PCR. Relative mRNA expression is shown as a ratio relative to *β-actin* and reported as means ± standard error of the mean (*n* = 6). ^a^*P* < 0.05 versus the control group; ^b^*P* < 0.05 versus the HFD group. (c) The effect of PS-ALA on protein expression of *Grp78* and *Chop* in the liver was assayed by western blotting, with *β-actin* as a loading control. Representative images of at least three independent experiments are shown. Protein expression of (d) *Grp78* and (e) *Chop* is presented as fold change relative to the control. Each bar denotes the mean ± standard error of the mean (*n* = 6). ^a^*P* < 0.05 versus the control group; ^b^*P* < 0.05 versus the HFD group.

To investigate the possible molecular mechanism underlying the beneficial effect of PS-ALA on NAFLD, gene and protein expressions of glucose-regulated protein 78 (*Grp78*) and C/EBP homologous protein (*Chop*), two markers of ER stress, were assayed. As the results shown in Figure 2, compared with the control, the protein and mRNA expressions of *Grp78* and *Chop* were statistically increased in HFD-fed mice, and the high levels were markedly inhibited by PS-ALA treatment (*P* < 0.05).

### 3.4. PS-ALA Inhibited Sterol Regulatory Element-Binding Protein (*Srebp*) Pathway Activation during ER Stress and Improved Lipid Metabolism

**Figure 3:**
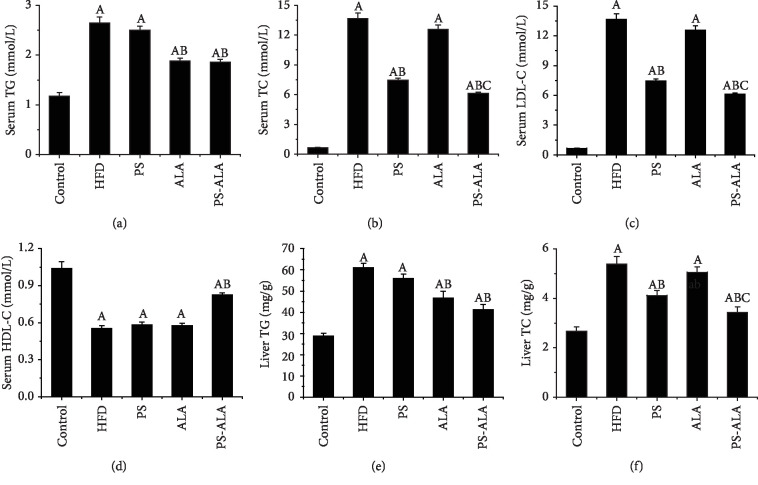
PS-ALA improves lipid profiles in mice. (a) Serum TG, (b) serum TC, (c) serum LDL-C, (d) serum HDL-C, (e) liver TG, and (f) liver TC. Each bar or point denotes the mean ± standard error of the mean (*n* = 10). ^a^*P* < 0.05 versus the control group; ^b^*P* < 0.05 versus the HFD group; ^c^*P* < 0.05 versus the PS group.

In the present study, HFD caused lipid disorder as shown by increased serum levels of TG, TC, and LDL-C and decreased HDL-C (*P* < 0.05). Both interventions of ALA and PS-ALA have an effect in alleviating the rise of TG and TC in serum and liver while a much lower level of TC was observed in the PS-ALA group (*P* < 0.05). Although PS treatment decreased serum TC and LDL-C, as well as, TC in the liver (*P* < 0.05), it had no effect on TG and HDL-C (*P* > 0.05). PS-ALA treatment overall improved lipid profiles (*P* < 0.05). These results implied that PS-ALA had a much better effect on ameliorating HFD-induced lipid disorder than ALA and PS (Figure 3).

**Figure 4:**
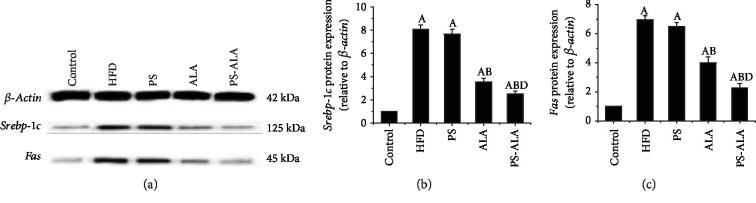
PS-ALA reduces protein expression of gene involved in de novo fat synthesis. (a) The effect of PS-ALA on protein expression of *Srebp-1c* and *Fas* in the liver was assayed by western blotting, with *β-actin* as a loading control. Representative images of at least three independent experiments are shown. Protein expression of (b) *Srebp-1c* and (c) *Fas* is presented as fold change relative to the control. Each bar denotes the mean ± standard error of the mean (*n* = 6). ^a^*P* < 0.05 versus the control group; ^b^*P* < 0.05 versus the HFD group; ^c^*P* < 0.05 versus the ALA group.

**Figure 5:**
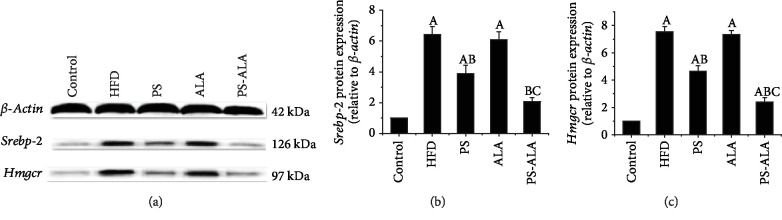
PS-ALA decreases protein expression of gene involved in cholesterol synthesis. (a) The effect of PS-ALA on protein expression of *Srebp-2* and *Hmgcr* in the liver was assayed by western blotting, with *β-actin* as a loading control. Representative images of at least three independent experiments are shown. Protein expression of (b) *Srebp-2* and (c) *Hmgcr* is presented as fold change relative to the control. Each bar denotes the mean ± standard error of the mean (*n* = 6). ^a^*P* < 0.05 versus the control group; ^b^*P* < 0.05 versus the HFD group; ^c^*P* < 0.05 versus the PS group.

To further explore the underlying mechanism by which PS-ALA modulated lipid metabolism, the protein expression of the crucial genes involved in TG and cholesterol synthesis during ER stress was detected. Results showed that PS-ALA supplement but not PS prominently lowered HFD-induced high protein expression of sterol regulatory element-binding protein-1c (*Srebp-1c*) and fatty acid synthase (*Fas*), which regulated de novo fat synthesis (*P* < 0.05) (Figure 4). Dietary PS significantly downregulated sterol regulatory element-binding protein-2 (*Srebp-2*) and 3-hydroxy-3-methylglutaryl-coenzyme A reductase (*Hmgcr*), which took charge of cholesterol synthesis in the liver, while a more pronounced effect was achieved by PS-ALA treatment (*P* < 0.05) (Figure 5). The results suggested the ameliorating effect of PS-ALA on lipid metabolism at least partly through inhibiting *Srebps* activation during ER stress.

### 3.5. PS-ALA Reduced Inflammatory Cytokines and Transaminase

**Figure 6:**
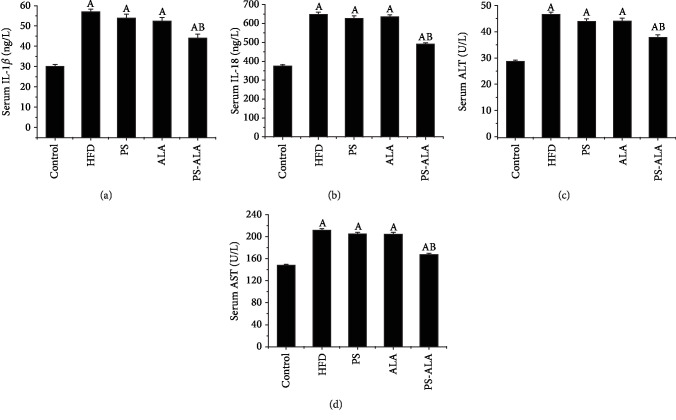
PS-ALA alleviates serum inflammatory cytokines and transaminases in mice: (a) serum IL-1*β*, (b) serum IL-18, (c) serum ALT, and (d) serum AST. Each bar denotes the mean ± standard error of the mean (*n* = 10). ^a^*P* < 0.05 versus the control group; ^b^*P* < 0.05 versus the HFD group.

Long-time HFD resulted in serious liver damage marked by high levels of serum IL-1*β*, IL-18, ALT, and AST (*P* < 0.05), while PS-ALA but not ALA or PS intervention significantly lowered all the inflammatory cytokines and transaminase mentioned above (*P* < 0.05), which suggest a synergistic effect. These results suggested that PS-ALA was capable of inhibiting HFD-induced inflammation and attenuating liver injury (Figure 6).

### 3.6. PS-ALA Weakened Inositol-Requiring 1*α* (*Ire1α*)/x-Box-Binding protein1s (*Xbp1s*) Pathway and Inflammasome Activation Induced by ER Stress

**Figure 7:**
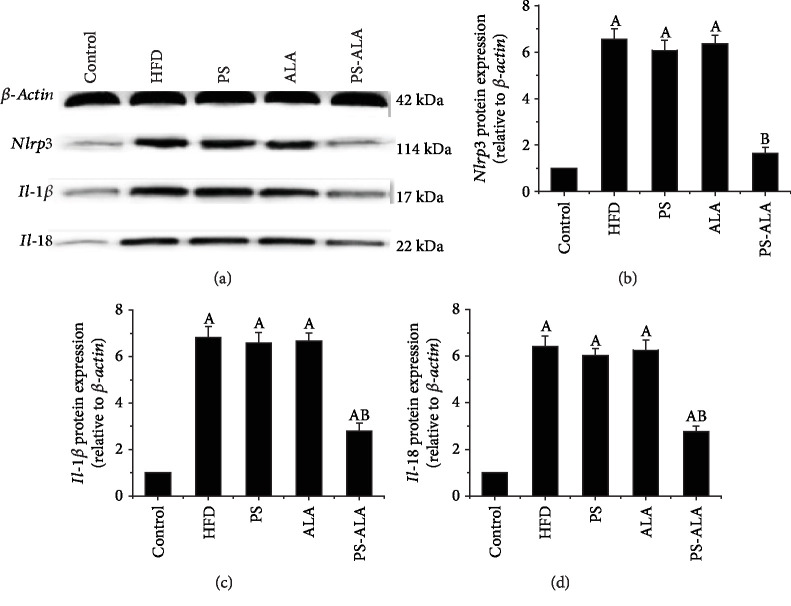
PS-ALA ameliorates inflammasome activation and proinflammatory cytokines in mice. (a) The effect of PS-ALA on protein expression of *Nlrp3*, *Il-1β*, and *Il-18* in the liver was assayed by western blotting, with *β-actin* as a loading control. Representative images of at least three independent experiments are shown. Protein expression of (b) *Nlrp3*, (c) *Il-1β*, and (d) *Il-18* is presented as fold change relative to the control. Each bar denotes the mean ± standard error of the mean (*n* = 6). ^a^*P* < 0.05 versus the control group; ^b^*P* < 0.05 versus the HFD group.

Compared with the control group, the protein expression of NOD-like receptor family pyrin domain-containing 3 (*Nlrp3*), *Il-1β*, and *Il-18* in the liver was higher in the HFD group, but the changes were diminished by PS-ALA treatment (*P* < 0.05) (Figure 7).

**Figure 8:**
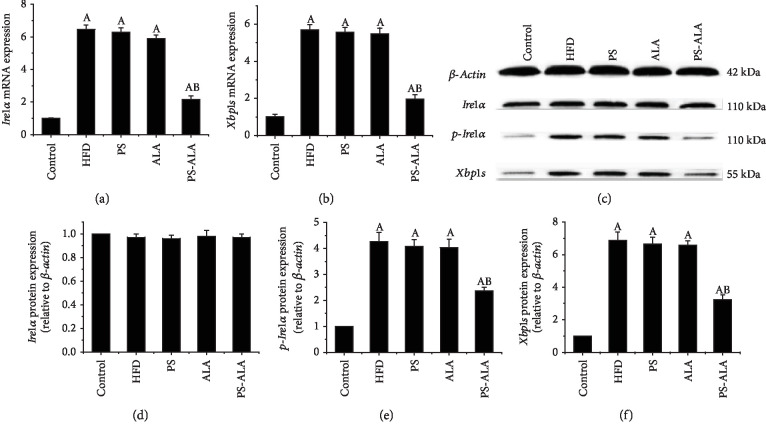
PS-ALA inhibits mRNA and protein expression of phosphorylated *Ire1α*, total *Ire1α*, and *Xbp1s* in mice. Effect of PS-ALA on mRNA expression of (a) *Ire1α* and (b) *Xbp1s* was assayed with quantitative real-time RT-PCR. Relative mRNA expression is shown as a ratio relative to *β-actin* and reported as means ± standard error of the mean (*n* = 6). ^a^*P* < 0.05 versus the control group; ^b^*P* < 0.05 versus the HFD group. (c) The effect of PS-ALA on protein expression of phosphorylated *Ire1α*, total *Ire1α*, and *Xbp1s* was assayed by western blotting, with *β-actin* as a loading control. Representative images of at least three independent experiments are shown. Protein expression of (d) phosphorylated *Ire1α*, (e) total *Ire1α*, and (f) *Xbp1s* is presented as fold change relative to the control. Each bar denotes the mean ± standard error of the mean (*n* = 6). ^a^*P* < 0.05 versus the control group; ^b^*P* < 0.05 versus the HFD group.

To further explore the cause for inflammation during ER stress and the protective action displayed by PS-ALA, gene and protein expressions of *Ire1α*/*Xbp1s* pathway that activated *Nlrp3* during ER stress were assessed. The result showed that mice feeding on HFD exhibited dramatically increased mRNA and protein levels of *Ire1α* and *Xbp1s* as compared with the control (*P* < 0.05), while the significant lower effect was achieved by PS-ALA treatment (*P* < 0.05). Moreover, the data clearly showed that PS-ALA treatment reversed the augmented phosphorylation activation of *Ire1α* and protein expression of *Xbp1s* (*P* < 0.05) (Figure 8). These data indicate that anti-inflammatory effect of PS-ALA may partly through inhibiting *Ire1α*/*Xbp1s* signal pathway and NLRP3 activation.

### 3.7. PS-ALA Attenuated ER Stress-Induced Oxidative Stress and Mitochondrial Dysfunction

**Figure 9:**
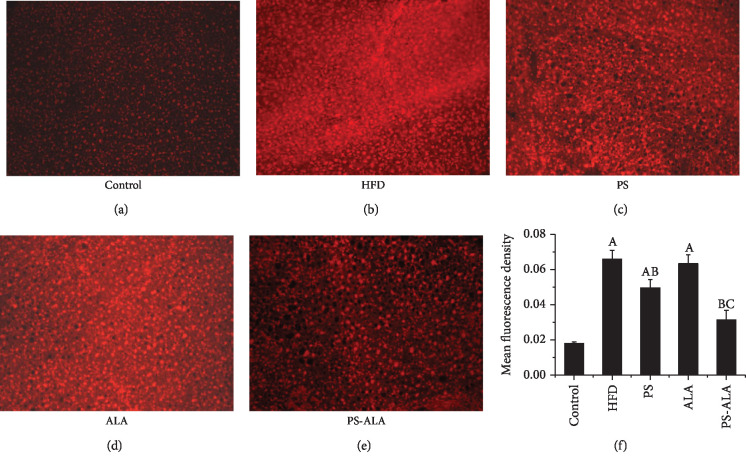
PS-ALA reduces hepatic ROS production of mice. (a) ROS in the liver of the mice was detected by using DHE which reacts with ROS and forms ETH that binds to DNA and produces a red fluorescence signal, visualized with a fluorescence microscope (×200) and quantified. (b) Fluorescence intensities in randomly selected areas of the images were quantified by using the IPP image analysis software. Each bar denotes the mean ± standard error of the mean (*n* = 6). ^a^*P* < 0.05 versus the control group; ^b^*P* < 0.05 versus the HFD group; ^c^*P* < 0.05 versus the PS group.

**Figure 10:**
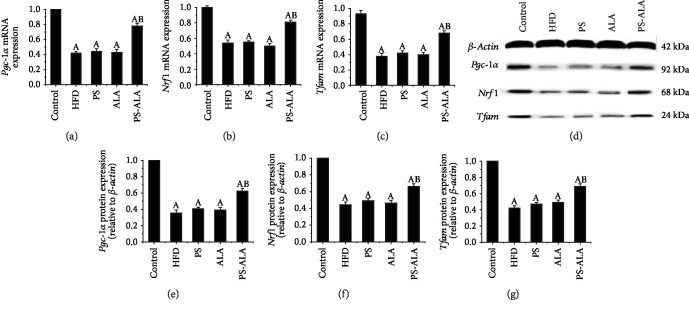
PS-ALA decreases mRNA and protein expression of *Pgc-1α*, *Nrf1*, and *Tfam* in mice. Effect of PS-ALA on mRNA expression of (a) *Pgc-1α*, (b) *Nrf1*, and (c) *Tfam* was assayed with quantitative real-time RT-PCR. Relative mRNA expression is shown as a ratio relative to *β-actin* and reported as means ± standard error of the mean (*n* = 6). *P* < 0.05 versus the control group; ^b^*P* < 0.05 versus the HFD group. (d) The effect of PS-ALA on protein expression of *Pgc-1α*, *Nrf1*, and Tfam was assayed by western blotting, with *β-actin* as a loading control. Representative images of at least three independent experiments are shown. Protein expression of (e) *Pgc-1α*, (f) *Nrf1*, and (g) *Tfam* is presented as fold change relative to the control. Each bar denotes the mean ± standard error of the mean (*n* = 6). ^a^*P* < 0.05 versus the control group; ^b^*P* < 0.05 versus the HFD group.

**Figure 11:**
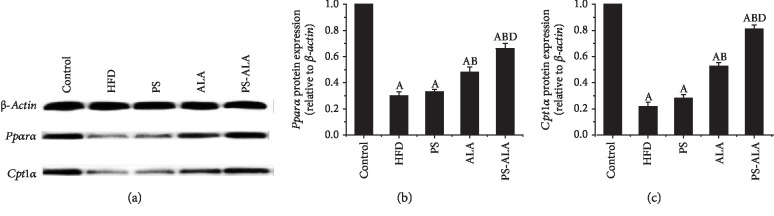
PS-ALA increases protein expression of genes involved in *β*-oxidation of fatty acids. (a) The effect of PS-ALA on protein expression of *Pparα* and *Cpt1a* was assayed by western blotting, with *β-actin* as a loading control. Representative images of at least three independent experiments are shown. Protein expression of (b) *Pparα* and (c) *Cpt1a* is presented as fold change relative to the control. Each bar denotes the mean ± standard error of the mean (*n* = 6). ^a^*P* < 0.05 versus the control group; ^b^*P* < 0.05 versus the HFD group; ^c^*P* < 0.05 versus the ALA group.

It has been reported that ER stress caused excessive production of ROS, which contributed to mitochondrial dysfunction and FA *β*-oxidation impairment. As the result illustrated in Figure 9, excessive ROS production in the liver was dramatically reduced after exposure to PS-ALA (*P* < 0.05). Also, PS-ALA intervention significantly improved mitochondrial biogenesis as exhibited by the higher mRNA and protein expression of peroxisome proliferator-activated receptor coactivator-1*α* (*Pgc-1α*), nuclear respiratory factor-1 (*Nrf1*), and transcription factor A mitochondria (*Tfam*) compared with HFD treatment (*P* < 0.05) (Figure 10). To further evaluate the mitochondrial oxidative capacity and explore the explanation of lower TG level in PS-ALA-treated mice, peroxisome proliferator-activated receptor alpha (*Pparα*) and carnitine palmitoyltransferase 1A (*Cpt1a*), the key genes involved in mitochondrial *β*-oxidation of FA, were assayed. Results showed that PS-ALA was more valid than ALA in increased mRNA and protein expression of *Pparα* and *Cpt1a* inhibited by HFD (*P* < 0.05) (Figure 11). These data indicated that PS-ALA reduced ROS production and protected the mitochondrion against oxidative injury, which in turn promoted FA *β*-oxidation and diminished TG accumulation.

## 4. Discussion

NAFLD is considered to be the clinical feature of metabolic syndrome and is associated with an increased risk of both prevalent and incident cardiovascular diseases, diabetes, and mellitus chronic kidney disease (CKD) [19]. Currently, no pharmacology is identified for NAFLD treatment, and a healthy diet and regular physical exercise represent the recommended treatment [20]. In recent years, numerous researches have focused on natural products or plant chemicals with lipid modulation, antioxidation, and anti-inflammation effects. This study demonstrated that PS-ALA was capable of ameliorating hepatic steatosis as well as optimizing lipid profiles, weakening inflammation, mitigating liver damage, and inhibiting oxidative stress induced by chronic lard-based HFD, which implied that PS attaching ALA had a good effect on protecting against NAFLD. Our results were supported by existing evidence showing that ALA prevented hepatic steatosis in HFD-feeding mice [21] and intake of *β*-sitosterol and stigmasterol alleviated NAFLD in western-style HFD-feeding mice [22].

It has been published that ER dysfunction was related to lipid disorder, inflammation, and oxidative stress during the development of NAFLD [23]. Also, a line of evidence announced the links between ER stress and NAFLD [24, 25]. Thus, in this work, we explored the underlying mechanism by which PS-ALA exerted NAFLD protection effect focusing on ER stress. ER stress was demonstrated to involve in the development of NAFLD, as evidenced by the findings that the patients with NASH generally expressed a high level of ER stress indicators, including *GRP78* and *CHOP* [26]. A vitriol study conducted in primary rat hepatocytes showed that cotreatment with ALA reversed the increased levels of *Grp78* and *Chop* [27]. Consistently, in this study, the increased mRNA and protein expression of *Grp78* and *Chop* induced by HFD was remarkably lowered by PS-ALA treatment. Thus, the protecting effect of PS-ALA on NAFLD may be partly through attenuating ER stress.

In parallel with liver steatosis induced by increased de novo lipogenesis (DNL), decreased FA *β*-oxidation, and reduced VLDL secretion, and increased uptake of circulating FA derived from the or diet, hepatic lipids accumulate can also as a result of ER stress [28]. Human studies revealed that *DNL* contributes to about a quarter of liver lipids in NAFLD patients [29]. Besides secreting membrane proteins, ER is also the major site of lipid synthesis in hepatocyte. In fact, DNL is mainly regulated by ER membrane-localized transcription factors, *Srebp-1c* for FA synthesis and S*rebp-2* for cholesterol synthesis [30]. A great deal of evidence has shown that chronic ER stress upregulated *Srebps* directly or indirectly, promoting TG and cholesterol synthesis and storage, which contributed to hepatic steatosis development [31, 32]. A previous study showed that ALA downregulated TG and cholesterol biosynthesis pathway by suppressing *Srebp-1c* and S*rebp-2* protein expression [33]. We found similar results with existing evidence that PS-ALA treatment lowered protein expression of *Srebp-1c* and *Fas* regulating de novo fat synthesis, as well as S*rebp-2* and *Hmgcr* taking charge of cholesterol synthesis. These data suggested the ameliorating effects of PS-ALA on lipid metabolism at least partly through inhibiting *Srebps* activation during ER stress.

When ER stress was induced by excessive SFAs, cholesterol, and misfolded proteins, UPR was triggered through activation of 3 pathways, controlled by IRE1a, PRK-like ER kinase (PERK), and transcription factor-6 (ATF6), to initiate an adaptive program [34]. However, if ER homeostasis is not restored by activating UPR recovery pathways, improper responses to ER stress will result in lipid disorder, inflammation, oxidative stress, and apoptosis, which may create a lipotoxic environment upon NAFLD [35–37]. It has been well known that chronic ER stress led to inflammation via activating the IRE1a signal pathway. Unfolded proteins in the ER lumen induced the endoribonuclease activity of IRE1a, which in turn spliced XBP1 mRNA to its mature form for translation into the transcription factor XBP1s. As a result, NLRP3 inflammasome was activated to cleave procaspase-1 to its active form, which causes maturation and secretion of IL-1*β* and IL-18 and induces metabolic inflammation [38]. A study worked by Lebeaupin et al. showed the prolonged and irremediable ER stress-induced immoderate *Ire1a* activation in livers of obese mice, which, in turn, activated the *Nlrp3* and subsequently initiated liver injury [39]. Also, plenty of studies suggest that NLRP3 is harmful to hepatic steatosis and NASH pathogenesis. Indeed, evidence from the clinical study showed that NAFLD patients had elevated protein expression of *NLRP3*, *IL-1β*, and *IL-18* [40]. In the animal module, NLRP3 deficiency protects against HFD-induced liver steatosis in mice [41]. These findings raise the possibility that suppression of NLRP3 inflammasome activation induced by IRE1a may be beneficial to the treatment of NAFLD. Recently, it has been published that metabolites of ALA mediated anti-inflammatory effects by inactivating *Nlrp3* inflammasome [42]. Meanwhile, dietary ALA supplementation was demonstrated to be effective in preventing hepatic steatosis, which is associated with inflammation and ER stress [21]. Flaxseed oil rich in ALA intervention apparently decreased the serum level of inflammatory cytokines (IL-6, IL-1*β*, MCP-1, and TNF-*α*) and attenuated hepatic steatosis by regulating ER stress [43]. Currently, Javanmardi et al. demonstrated that intake of 1.6 g/d PS efficiently not only lowered LDL-C but also decreased TNF-*α*, AST, and ALT in patients with NAFLD [44]. It was also reported that the liver sitosterol ratio to cholesterol negatively associated with hepatic steatosis and inflammation in obese individuals with NAFLD [45]. In accordance with these results, in this NAFLD model, serum concentrates and protein expressions of the *Nlrp3* inflammasome, *Il-1β*, and *Il-18* were elevated by HFD, along with phosphorylation activation of *Ire1a* and increased mRNA and protein expression of *Xbp1s*. PS-ALA prevented those responses, which indicated that the anti-inflammatory effect of PS-ALA may link to inhibiting *Ire1a*/*Xbp1s* pathway and *Nlrp3* inflammasome activation.

It has been well documented that chronic ER stress initiated oxidative stress via augmenting ER Ca^2+^ release and oxidoreductin-1 activity, both of which resulted in mitochondrial damage [46]. *β*-Oxidation mainly proceeding in mitochondria is the primary pathway for FA decomposition. Mitochondrial dysfunction is concurrent with suboptimal or incomplete fat oxidation, causing accumulation of TG, which can bring about hepatic steatosis [47]. In human and rodent models with NAFLD, mitochondrial impairment is a central feature of SFL to NASH transition [48]. Hence, improving mitochondrial function may be a potential therapeutic target for NAFLD. It has been well established that the upregulation of proteins took charge in mitochondrial biogenesis could enhance oxidative metabolic capacity. Nrf1 is a transcription factor that regulates nuclear gene coding for mitochondrial respiratory chain proteins and can be activated by PGC-1*α*, which accelerates the transcription of genes involved in oxidative phosphorylation. PGC-1*α* and Nrf1 coactivate Tfam maintaining and regulating mtDNA transcription and replication [49]. In the present work, our data show that HFD resulted in overproduction of ROS, which impaired mitochondrial function marked by lower gene and protein expressions of *Pgc-1α*, *Nrf1*, and *Tfam*. PS-ALA treatment not only remarkably reduced ROS production but also notably enhanced transcripts and expressions of *Pgc-1α*, *Nrf1*, and *Tfam*. Moreover, dysfunctional mitochondria that led to reduction of protein expression of *Pparα* and *Cpt1a*, two important regulators administering FA *β*-oxidation, was prevented by PS-ALA supplementation. Others suggested that beta-sitosterol repressed acute hepatic injury by inhibiting oxidative stress in mice [50]. Evidence from a clinical study showed that dietary 0.945 g/d n-3 PUFA (21% eicosapentaenoic (EPA), 16% docosahexaenoic (DHA) acids, and 64% ALA) for 6 months significantly improved hepatic lipogenesis, ER stress, and mitochondrial function in patients with NASH [51]. Our previous study proved that treatment with ALA rich in flaxseed oil significantly increased gene and protein expressions of *Pparα* and key enzymes in FA *β*-oxidation (*Cpt1a* and *Acox1*), which in turn reduced TG accumulation in the liver [52]. Our findings together with these others suggest that PS-ALA may inhibit ROS production and improve mitochondrial biogenesis, thus promoting FA *β*-oxidation and substantially reducing fat accumulation in the liver.

## 5. Conclusions

PS-ALA has a good effect in suppressing hepatic steatosis, attenuating inflammatory response, and inhibiting oxidative stress in an HFD-induced NAFLD mouse model. The underlying mechanism may be that PS-ALA blocks *Srebps* activation, *Ire1a*/*Xbp1s* signal pathway sensitization, and ROS overproduction by rescuing the adaption to ER stress, leading to decreased lipid accumulation, wakened *Nlrp3* activity, and improved mitochondrial biogenesis. The results suggest that PS-ALA may be a novel candidate for NAFLD prevention and treatment.

## Figures and Tables

**Table 1:** Composition of the experimental diets (g).

Ingredient	Control group	HFD group	PS group	ALA group	PS-ALA group
Casein	200	200	200	200	200
L-Cystine	3	3	3	3	3
Corn starch	315	72.8	72.8	72.8	72.8
Maltodextrin 10	35	100	100	100	100
Sucrose	350	172.8	172.8	172.8	172.8
Cellulose, BW200	50	50	50	50	50
Soybean oil	25	25	25	25	25
Lard	20	177.5	159.9	166.1	148.5
Mineral mix S0026	10	10	10	10	10
Dicalcium phosphate	13	13	13	13	13
Calcium carbonate	5.5	5.5	5.5	5.5	5.5
Potassium citrate, 1 H_2_O	16.5	16.5	16.5	16.5	16.5
Vitamin mix V 10001	10	10	10	10	10
Choline bitartrate	2	2	2	2	2
Cholesterol	0	20	20	20	20
PS (g)	0	0	17.6	0	0
ALA (g)	0	0	0	11.4	0
PS-ALA (g)	0	0	0	0	29.0
Total (g)	1055	878.1	878.1	878.1	878.1
Energy (kcal)	4057	4057	4057	4057	4057

**Table 2:** Primer sequences used for real-time PCR.

Gene	Forward primer	Reverse primer
*Grp78*	5′-CCTGCGTCGGTGTGTTCAA-3′	5′-ATCGCCAATCAGACGCTCC-3′
*Chop*	5′-GCCTTTCACCTTGGAGACGG-3′	5′-GGACGCAGGGTCAAGAGTAGTG-3′
*Ire1α*	5′-ACACTGCCTGAGACCTTGTTG-3′	5′-GGAGCCCGTCCTCTTGCTA-3′
*Xbp1s*	5′-CTGAGTCCGAATCAGGTGCAG-3′	5′-GTCCATGGGAAGATGTTCTGG-3′
*Pgc-1α*	5′-TATGGAGTGACATAGAGTGTGCT-3′	5′-CCACTTCAATCCACCCAGAAAG-3′
*Nrf1*	5′-GACCTTGCCACAGGCAGGTAA-3′	5′-CGCCTGCTCCATGAACACTC-3′
*Tfam*	5′-TCAGGAGCAGCAGGCACTACA-3′	5′-CTGAGCTCCGAGTCCTTGAACAC-3′
*β-Actin*	5′CATCCGTAAAGACCTCTATGCCAAC-3′	5′-ATGGAGCCACCGATCCACA-3′

**Table 3:** Effect of PS-ALA on food intake, body weight, and liver weight/body weight in mice throughout the feeding period.

Parameters	Groups				
	Control	HFD	PS	ALA	PS-ALA
Food intake (g/d)	4.04 ± 0.06	4.07 ± 0.10	3.97 ± 0.09	4.00 ± 0.13	4.01 ± 0.11
Energy consumption (kcal/d)	15.57 ± 0.24	19.26 ± 0.47^a^	18.82 ± 0.40^a^	18.94 ± 0.61^a^	18.97 ± 0.50^a^
Initial body weight (g)	21.91 ± 0.47	22.12 ± 0.64	22.11 ± 0.59	22.20 ± 0.46	21.44 ± 0.47
Final body weight (g)	29.07 ± 0.76	39.54 ± 1.43^a^	36.95 ± 1.71^a^	37.25 ± 0.84^a^	35.08 ± 1.71^ab^
Liver weight (g/100 g body weight)	3.37 ± 0.11	4.72 ± 0.09^a^	4.65 ± 0.09^a^	4.58 ± 0.10^a^	3.73 ± 0.15^ab^

Values are given as means ± standard error of the mean (*n* = 10). ^a^*P* < 0.05 versus the control group; ^b^*P* < 0.05 versus the HFD group.

## Data Availability

The data used to support the findings of this study are available from the corresponding authors upon request.
